# Effectiveness of omega-3 fatty acid supplementation for pruritus in patients undergoing hemodialysis

**DOI:** 10.3389/fnut.2024.1328469

**Published:** 2024-01-25

**Authors:** Alireza Rafieipoor, Mahdie Torkaman, Fatemeh Azaryan, Aryan Tavakoli, Mohammad Keshavarz Mohammadian, Atefeh Kohansal, Hanieh Shafaei, Pouya Mirzaee, Zeinab Motiee Bijarpasi, Parsa Bahmani, Masoud Khosravi, Saeid Doaei, Maryam Gholamalizadeh

**Affiliations:** ^1^Master of Nursing, Hormozgan University of Medical Sciences, Bandar Abbas, Hormozgan, Iran; ^2^Department of Chemical Engineering, Science and Research Branch, Islamic Azad University, Tehran, Iran; ^3^Department of Physiology, School of Medicine, Zahedan University of Medical Sciences, Zahedan, Iran; ^4^Department of Clinical Nutrition, School of Nutritional Sciences and Dietetics, Tehran University of Medical Sciences, Tehran, Iran; ^5^Department of Nutrition, Science and Research Branch, Islamic Azad University, Tehran, Iran; ^6^School of Nutrition and Food Sciences, Shiraz University of Medical Sciences, Shiraz, Iran; ^7^School of Nursing and Midwifery, Guilan University of Medical Sciences, Rasht, Iran; ^8^Department of Medicine, Faculty of Medicine, Semnan University of Medical Sciences, Semnan, Iran; ^9^School of Health, Guilan University of Medical Sciences, Rasht, Iran; ^10^Department of Nutrition, Shahid Beheshti University of Medical Sciences, Tehran, Iran; ^11^Urology Research Center, Razi Hospital, School of Medicine, Guilan University of Medical Sciences, Rasht, Iran; ^12^Department of Community Nutrition, Faculty of Nutrition and Food Technology, National Nutrition and Food Technology Research Institute, Shahid Beheshti University of Medical Sciences, Tehran, Iran; ^13^Cancer Research Center, Shahid Beheshti University of Medical Sciences, Tehran, Iran

**Keywords:** chronic kidney disease, omega-3 fatty acids, pruritus, dialysis, CKD

## Abstract

**Background:**

Patients undergoing hemodialysis (HD) frequently experience the chronic kidney disease-associated pruritus (CKD-aP).

**Objective:**

The aim of this study was to investigate the effectiveness of omega-3 supplementation in the management of CKD-aP in patients undergoing hemodialysis.

**Methods:**

In this triple blind, randomized clinical trial, the effect of the omega-3 supplement on uremic CKD-aP was assessed in 112 chronic hemodialysis patients at Caspian Hemodialysis Center in Rasht, Iran. Patients were randomly divided into the intervention group receiving omega-3 supplements (3 g/day) and the control group receiving placebo containing MCT oil for 2 months.

**Results:**

Omega-3 supplementation had no effect on CKD-aP. The results did not change after adjusting for age and sex, additional adjustments for weight, height, physical activity, smoking, and alcohol use, additional adjustments for underlying diseases and weight, height, physical activity, smoking, and drinking alcohol, and further adjustments for underlying diseases and biochemical indices.

**Discussion:**

Omega-3 supplementation for 2 months had no effect on CKD-aP in patients with CKD. Further studies with longer duration are warranted.

**Clinical Trial Registration:**

https://www.irct.ir/trial/66638, IRCT20151226025699N6

## Graphical Abstract

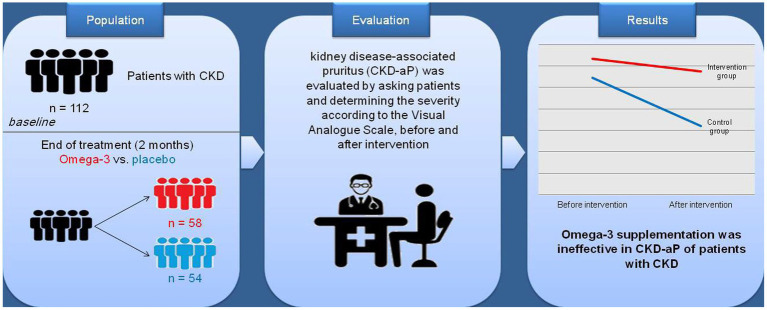

## Introduction

Chronic kidney disease-associated pruritus (CKD-aP), also previously known as uremic pruritus, is a common troublesome symptom in patients with advanced CKD or end-stage renal disease (ESRD) (1, 2). This complaint is most commonly described as daily or near-daily CKD-aP in large, symmetrical areas and is observed in up to 60% of dialysis patients (2–4) and affects 15–49% of pre-dialysis CKD patients and 50–90% of those undergoing dialysis, including peritoneal dialysis and hemodialysis (HD) (5).

The exact cause of pruritus in HD patients is not fully understood, but is believed to be multifactorial. Possible risk factors for uremic CKD-aP in HD patients are age, sex, calcium phosphate imbalance, prolonged dialysis duration, secondary hyperparathyroidism, concomitant cardiovascular diseases, heart failure, pulmonary diseases, liver diseases, and neurological diseases (6). Risk factors for developing pruritus in non-dialysis CKD patients include older age, female gender, advanced stage of CKD, lung disease, diabetes, and depression (1). CKD-aP dramatically influences the quality of life, so that about half of CKD patients experience from CKD-aP throughout the day and one third of them are most affected at night which causes sleep disturbances and depression (7). In particular, irritating symptoms of CKD-aP not only affect quality of life, but also lead to poor medical outcomes and patients with severe CKD-aP are also more likely to abandon or miss dialysis sessions (8). CKD-aP is also associated with worse clinical outcomes such as mortality, increased medication use (intravenous antibiotics, erythropoiesis-stimulating drugs, and iron supplementation), higher rate of infections, and hospitalizations (8–10).

Although the definite mechanisms for uremic CKD-aP have not been determined, some pathologic mechanisms are proposed for uremic CKD-aP such as increasing oxidants and inflammatory processes, loss of serum anti-oxidants in CKD patients during HD, imbalance of ions and electrolytes, and inability to excrete nitrogen products and other waste materials (11). Recent studies suggested using anti-inflammatory and antioxidant agents could lead to reduce pruritus (12). Several small studies have examined different dietary interventions against pruritus, but the efficacy and optimal treatment of these interventions are not yet well defined (13, 14).

Chronic kidney disease-associated pruritus may be influenced by essential fatty acids and their metabolites involved in the cyclooxygenase and lipoxygenase pathways, including prostaglandins and leukotrienes, respectively (15). Omega-3 fatty acid supplementation may provide many health benefits to dialysis patients by modulating the structure and function of cell membranes and the synthesis of lipid mediators such as eicosanoids. Omega-3 fatty acids have a key role in improving a variety of human body processes, including inflammatory and immune processes, atherosclerosis and cardiovascular diseases, arrhythmias, rheology, blood pressure, and lipid regulation (16). However, the effect of omega-3 fatty acids on CKD-aP in CKD patients is not clear. So, this study attempted to investigate the effectiveness of omega-3 fatty acids supplement for CKD-aP in HD patients undergoing dialysis.

## Methods

### Study design and participants

A randomized controlled triple blind trial was conducted on patients with CKD treated with HD in 2022–2023 at Caspian Hemodialysis Center in Rasht, Iran. A randomized block sampling method and the WinPepi program were used to assign the participants to the intervention and control groups. Finally, 16 blocks were determined and in each block, three persons were assigned in the control group and three persons were assigned in the intervention group in a random sequence. The intervention and control groups were randomly assigned using a web-based software.^1^ Both groups were matched in terms of age and sex. This study was a triple blind clinical trial which ensures that neither the patients, the researchers, nor the statistical analyst are aware of the study components.

Inclusion criteria were completing written consent form, age over 20 years, KT/V (liters/min) higher than the standard range, no consumption of omega-3 fatty acid supplement during the last 3 months before starting the study, no history of peritoneal dialysis, no surgery in the previous 6 months, no history of hypersensitivity response to omega-3 fatty acid supplementation and/or medium chain triglycerides (MCTs) oil, no history of allergy to fish and fish products, and not to be pregnant. Exclusion criteria were refusal to continue the participation in the study, diagnosis of psychiatric conditions and intellectual disability, have active inflammatory, infection, pulmonary, cardiac, hemoglobinopathies and coagulopathy conditions, which may interfere with the research process, malignancy, and recent use of immunosuppressant, chemotherapeutic or anticoagulant drugs such as warfarin and the use of nonsteroidal anti-inflammatory medications, corticosteroids, incomplete medical documents, non-compliance with omega-3 supplementation program, patients not disciple to the hemodialysis program, disease aggravation, and the need for hospitalization and surgery.

Data on the demographic and socioeconomic status of individuals were collected using the medical records and face to face interviews. Also, medical information such as medical history, dialysis sessions per week, drug history, blood pressure, and serum biochemical indices including hemoglobin (HGB), hematocrit (HCT), and platelet count (PLT), were collected from the patients’ files. The participants’ height and weight were measured using a tape meter and a validated scale, respectively. The dialysis sheets were used to assess nutritional supplements received by the patients.

### The intervention

Three capsules of omega-3 fatty acids supplement including 3 g of omega-3 fatty acids was given daily to the intervention group [each capsule contained about 180 mg eicosapentaenoic acid (EPA) and 120 mg dosahexaenoic acid (DHA) and 700 mg other omega-3 fatty acids; Zahravi Pharmaceutical Co, Tabriz, Iran] were given orally daily for 2 months to the patients in the intervention group. Previous studies reported high bioavailability of the fatty acids (20% of available EPA and DHA is absorbed from fish oil supplements) (17), which can increase the amount of omega-3 fatty acids in the serum (18, 19). The control group received three placebo capsules containing medium-chain triglyceride (MCT) (Zahravi Pharmaceutical Co, Tabriz, Iran). The participants were supplied with 21 capsules on a weekly basis. Both intervention and placebo consumptions were followed up with the patients through regular phone calls.

### Pruritus measurements

Dialysis CKD-aP was evaluated by asking patients and after confirmation of CKD-aP diagnosis. The assessment of itch severity was performed by the Visual Analog Scale (the WI-NRS) (20). A score of 0–16 was considered as no CKD-aP and a score of 17–48 was considered as having pruritus.

### Statistical analysis

The Kolmogorov–Smirnov test was used to determine the normal distribution. T-test and Chi-squared test were used to compare the quantitative and qualitative data between two groups, respectively. The logistic regression method was used to determine the effect of omega-3 fatty acids supplementation on CKD-aP after adjusting the confounding variables including age, sex, weight, height, physical activity, smoking, drinking alcohol, underlying diseases, and biochemical indices in different models. SPSS version 20 was used for all statistical analysis and a probability level of *p* < 0.05 was considered to be statistically significant.

## Results

General characteristics of participants are presented in Table 1. Totally, 112 patients, with mean age of 61.17 ± 12.35 in the intervention group and 55.33 ± 12.6 in the placebo group were included (*p* > 0.05). All the demographic data or clinical characteristics at baseline were normally distributed and the groups were not significantly different. No significant difference was found between the groups regarding weight, height, amount of sleep, HGB, CBC, PLT, sex, marital status, history of diabetes, hypertension, metabolic disorder, heart disease, tobacco use, and alcohol use.

**Table 1:** The general characteristics of the participants.

	Intervention group (*N* = 58)	Placebo group (*N* = 54)	*p* ^*^
Age (years)	61.17 ± 12.35	55.33 ± 12.6	0.14
Weight (kg)	67.87 ± 13.64	71.21 ± 14.0	0.204
Height (cm)	164.28 ± 9.32	167.19 ± 9.02	0.094
Amount of sleep (h)	8.0 ± 2.51	7.96 ± 2.52	0.951
HGB	11.0 ± 1.53	11.46 ± 1.42	0.100
HCT	34.98 ± 4.75	36.34 ± 4.49	0.118
PLT	197.33 ± 75.18	205.92 ± 86.58	0.577
Sex			
Females, *n* (%)	28 (48.3)	17 (31.5)	0.084
Male, *n* (%)	30 (51.7)	37 (68.5)	0.08
Married, *n* (%)	53 (94.6)	48 (92.3)	0.71
Diabetes, *n* (%)	26 (43.3)	26 (47.3)	0.71
Hypertension, *n* (%)	48 (81.4)	41 (74.5)	0.49
Heart Disease, *n* (%)	16 (26.7)	14 (25.5)	1.00
Tobacco use, *n* (%)	4 (6.7)	8 (14.5)	0.23
Alcohol use, *n* (%)	0 (0.0)	1 (1.8)	0.47

^*^Obtained using independent *t*-test and qui-squared test for quantitative and qualitative variables, respectively. *p* < 0.05 was considered as significant. HGB, Hemoglobin; HCT, Hematocrit; and PLT, Platelet count.

The data in Table 2 show that no significant difference was found between the frequency of CKD-aP between the intervention and control groups before and after the intervention. Furthermore, decrease in the frequency of CKD-aP in the intervention group (31.7–28.8%) was not statistically significant compared to the placebo group (27.3–16%) (Table 3). The effect of supplementation with omega-3 on CKD-aP in CKD patients was not significant after adjustments for age and sex (Model 1) (OR = 0.72; 95% CI: 0.27–1.92; *p* = 0.51), additional adjustments for weight, height, physical activity, smoking, and drinking alcohol (OR = 0.9; 95% CI: 0.3–2.72; *p* = 0.87) (Model 2) and further adjustments for underlying diseases and biochemical indices (OR = 0.79; 95% CI: 0.24–2.54; *p* = 0.69) (Model 3).

**Table 2:** Frequency of pruritus among the control and intervention groups.

	Controls	Intervention	*p* ^*^
Pruritus before intervention	15 (27.3%)	19 (31.7%)	0.68
Pruritus after intervention	4 (16%)	15 (28.8%)	0.27
*p* ^*^	0.08	0.34	

^*^Obtained using qui-squared test. *p* < 0.05 was considered as significant.

**Table 3:** The association of omega-3 supplementation and pruritus in patients with CKD.

	OR (CI95%)	*p* ^*^
Model 1	0.72 (0.27–1.92)	0.51
Model 2	0.91 (0.3–2.72)	0.87
Model 3	0.79 (0.24–2.54)	0.69

^*^Obtained using logistic regression. Model 1: Adjusted for age and sex, Model 2: Further adjustments for weight, height, physical activity, smoking, and drinking alcohol, and Model 3: Additional adjustments for underlying diseases and biochemical indices.

## Discussion

In the present study, the effect of omega-3 supplementation on CKD-aP in HD patients was examined. The results showed that although the CKD-aP decreased after the intervention, there was no statistically significant difference between the intervention group and the placebo group after the intervention (Graphical abstract). Previous studies reported contradictory results on the efficacy of omega-3 fatty acid supplementation on CKD-aP in CKD patients. Several previous reports demonstrated the beneficial effects of omega-3 fatty acids supplementation in treatment of uremic CKD-aP in HD patients. For example, Ghanei et al. (13) by using omega-3 fatty acids in a double-blind randomized study found that CKD-aP was decreased up to 65% in HD patients suffering from pruritus. So it seems that omega-3 fatty acids could be used as an efficient strategy in the treatment of CKD-aP in uremic patients (13). In addition, Shayanpour et al. (14) concluded that the omega-3 supplement could reduce uremic pruritus in chronic HD patients. They found that the mean score of itching score in the intervention group decreased from 3.56 to 1.72 (*p* < 0.001) (14).

Moreover, in contrast with the present study, Begum et al. preformed a prospective, randomized, double-blinded, controlled study to compare the effects of the supplementations with fish oil, rich in omega-3 fatty acids, compared with safflower oil, rich in omega-6 fatty acids, on symptoms of CKD-aP in HD patients. The intervention group received six fish oil capsules (728 mg omega-3 fatty acids in each capsule) and the control group received six safflower oil capsules (704 mg omega-6 fatty acids in each capsule) per day for 16 weeks. The results showed that despite the absence of a significant difference in the mean baseline of CKD-aP score between two groups, the frequency of CKD-aP decreased in the fish oil group compared to the safflower oil group after 16 weeks (15). However, the duration of this intervention was almost twice as long as the present study and it is possible that anti CKD-aP effects of omega-3 fatty acids appear after long term supplementation. Some studies have shown that it takes at least 3 months for omega-3 fatty acids to exert their anti-inflammatory effects (21). Furthermore, in our study, MCT was used as a placebo for the control group, and at the end of the study, the rate of CKD-aP decreased in both groups. It is possible that MCT oil, similar to omega-3 fatty acids, has beneficial effects in reducing CKD-aP. However, there are few studies on the effects of MCT on CKD-aP. A recent study by Abbasi et al. (22) reported that coconut oil, which is a rich source of MCT, was effective on reducing pruritus in ESRD patients. Also, the anti-itching effects of omega-3 fatty acids may be different according to their origin (animal or vegetable) (23, 24) or the composition of their fatty acids (25).

The exact mechanisms of the possible effects of omega-3 fatty acids on CKD-aP is not yet determined. Dysregulation of immune system and chronic inflammation are among the potential contributors of the development of CKD-aP through numerous mediators including interleukin-2 (IL-2), prostaglandin E2 (PGE2), serotonin, histamine, proteases, and platelet activating factor. Anti-inflammatory and immune regulatory properties of the omega-3 fatty acids might be responsible for ameliorating CKD-aP (26). Other causes of the ineffectiveness of omega-3 fatty acids in CKD-aP is that other inflammatory mediators which are considered to be independent from the effects of omega-3 fatty acids (such as serotonin, histamine, protease, platelet-activating factor, etc.) may play a role in the clinical symptoms of CKD-aP. Moreover, CKD-aP may also be caused by non-inflammatory factors such as uremia, hyperparathyroidism, and calcium-phosphate imbalance (27).

However, the present study had some limitations. First, other predisposing factors for CKD-aP such as uremia and hyperparathyroidism were not controlled in this study. In addition, the duration of the intervention period was relatively short. Despite some study limitations, it should be noted that even mild relief of CKD-aP may have clinical significance in patients’ condition. Therefore, considering the many health benefits of omega-3 fatty acids for CKD and the negligible risk profile, omega-3 intake may be widely applicable for CKD patients.

## Conclusion

This randomized clinical trial did not support the efficacy of omega-3 fatty acid supplementation in CKD-aP of patients with CKD undergoing hemodialysis. Further clinical studies with different doses and types of omega-3 fatty acids, larger sample sizes, and longer durations along with the evaluation of potential pathophysiological pathways involved in CKD-aP are warranted to provide evidence-based recommendations and clinical guidelines regarding the effects of omega-3 fatty acids on CKD-aP.

## Data availability statement

The raw data supporting the conclusions of this article will be made available by the authors, without undue reservation.

## Ethics statement

This study was approved by the ethical committee of the cancer research center, Guilan University of Medical Sciences (code: IR.GUMS.REC.1401.307). The studies were conducted in accordance with the local legislation and institutional requirements. The participants provided their written informed consent to participate in this study.

## Author contributions

AR: Software, Writing – original draft. MT: Data curation, Writing – original draft. FA: Data curation, Writing – original draft. AT: Data curation, Writing – original draft. MM: Writing – original draft. AK: Data curation, Writing – original draft. HS: Data curation, Writing – review & editing. PB: Data curation, Writing – review & editing. MK: Project administration, Writing – original draft. SD: Methodology, Software, Writing – original draft. MG: Software, Writing – original draft. PM: Data curation, Writing – review & editing. ZM: Data collection, editing.

## Footnotes

^1^https://www.randomizer.org
